# Comparison of the Agronomic, Cytological, Grain Protein Characteristics, as Well as Transcriptomic Profile of Two Wheat Lines Derived From Wild Emmer

**DOI:** 10.3389/fgene.2021.804481

**Published:** 2022-01-28

**Authors:** Fangyi Gong, Tiangang Qi, Tian Zhang, Yusen Lu, Jia Liu, Xiaoying Zhong, Jingshu He, Yunfang Li, Youliang Zheng, Dengcai Liu, Lin Huang, Bihua Wu

**Affiliations:** ^1^ State Key Laboratory of Crop Gene Exploration and Utilization in Southwest China, Triticeae Research Institute, Sichuan Agricultural University, Chengdu, China; ^2^ Chengdu Institute of Biology, Chinese Academy of Science, Chengdu, China; ^3^ Key Laboratory of Crop Genetic Resources and Improvement, Ministry of Education, Sichuan Agricultural University, Chengdu, China

**Keywords:** wild emmer wheat, GPC, transcriptome, processing quality, gluten

## Abstract

Two advanced wheat lines BAd7-209 and BAd23-1 without the functional gene *GPC-B1* were obtained from a cross between common wheat cultivar Chuannong 16 (CN16) and wild emmer wheat accession D97 (D97). BAd7-209 showed superior quality parameters than those of BAd23-1 and CN16. We found that the components of glutenins and gliadins in BAd7-209 and BAd23-1 were similar, whereas BAd7-209 had higher amount of glutenins and gliadins than those of BAd23-1. RNA sequencing analysis on developing grains of BAd7-209 and BAd23-1 as well as their parents revealed 382 differentially expressed genes (DEGs) between the high–grain protein content (GPC) (D97 + BAd7-209) and the low-GPC (CN16 + BAd23-1) groups. DEGs were mainly associated with transcriptional regulation of the storage protein genes, protein processing in endoplasmic reticulum, and protein export pathways. The upregulated gluten genes and transcription factors (e.g., NAC, MYB, and bZIP) may contribute to the high GPC in BAd7-209. Our results provide insights into the potential regulation pathways underlying wheat grain protein accumulation and contribute to make use of wild emmer for wheat quality improvement.

## Introduction

Wheat (*Triticum aestivum* L.) is one of the most important staple crops, which can be processed into a wide range of products such as bread, noodle, and biscuit. Grain protein content (GPC) is an important quality trait in wheat and determines the nutritional value and processing quality (Shewry et al., 1995, 2009). However, GPC and grain yield-related traits are usually negative correlated (Groos et al., 2003), which hampered their simultaneous improvement in conventional wheat breeding program. The characterization and transferring of gene(s)/quantitative trait loci (QTLs) from wheat wild relatives is an effective strategy in the development of elite varieties with high GPC and/or yield (Krugman et al., 2018; Liu et al., 2019; Xiang et al., 2019; Gong et al., 2021).

Wild emmer (*T. turgidum* ssp. *dicoccoides*, 2n = 4x = 28, AABB), the tetraploid ancestor of common wheat, provides a valuable reservoir of genetic variation for GPC (Uauy et al., 2006; Liu et al., 2019). A number of QTLs affecting GPC were reported in wild emmer wheat (Joppa et al., 1997; Gonzalez-Hernandez et al., 2004; Uauy et al., 2006; Fatiukha et al., 2019; Liu et al., 2019). To date, however, only *Gpc-B1* on chromosome 6BS has been cloned. The introgression of *Gpc-B1* in wheat breeding programs can significantly improve GPC while reduce grain-yield related traits in some wheat lines and environments (Uauy et al., 2006; Chen et al., 2017). Therefore, it is desirable to identify GPC-QTLs that are not negative correlated or less correlated with grain yield-related traits.

GPC was regulated by a plethora of genes and easily affected by environment (Liu et al., 2019). In forward genetics, identification of candidate genes related to GPC is a time-consuming and laborious process. RNA sequencing (RNA-seq) provides high-resolution methods for deciphering quality traits and quantifying expression levels of candidate genes on a genome-wide scale (Furtado et al., 2015; Rangan et al., 2017). Currently, RNA-seq has been utilized to study the differentially expressed genes (DEGs) and regulation networks that associated with wheat grain protein accumulation (Cantu et al., 2011; Gong et al., 2021).

Grain protein synthesis in cereal crops were determined by several pathways, mainly including transcriptional regulation of the storage protein genes (glutenin and gliadin) and protein processing in endoplasmic reticulum (ER) and Golgi apparatus. Transcription factors (TFs) belong to bZIP, Dof, MYB, and NAC families have been widely reported in transcriptional regulation of grain protein genes in rice, maize, and barley (Suzuki et al., 1998; Onodera et al., 2001; Diaz et al., 2002; Shewry and Halford, 2002; Zhang et al., 2019). Previous studies demonstrated that the protein processing in ER and Golgi apparatus had crucial role in grain protein synthesis in rice (Takemoto et al., 2002; Wang et al., 2016; Ren et al., 2020), whereas their function for GPC was less reported in wheat.

In our previous studies, the agronomically stable advanced wheat lines were developed from a cross between common wheat cultivar Chuannong 16 (CN16) and wild emmer accession D97 (D97) followed by successive selfing (Jiang et al., 2017; Wang et al., 2018; Liu et al., 2019). Some advanced lines with simultaneous improvement of GPC and thousand-kernel weight (TKW) were obtained. In the present study, two sister lines, BAd7-209 and BAd23-1, with desirable agronomic traits were obtained from CN16×D97. These two lines showed contrasting GPC, while both of them did not contain the functional *Gpc-B1*. A comparison of the transcriptomes of developing grains from BAd7-209, BAd23-1, CN16, and D97 revealed candidate genes and regulation pathways that may be contributed to wheat grain protein accumulation.

## Materials and Methods

### Plant Materials

Two sister lines BAd7-209 and BAd23-1 as well as their parents D97 and CN16 were kept at the Triticeae Research Institute, Sichuan Agricultural University, Chengdu, China. CN16 and BAd23-1 were characterized as low-gluten wheat lines, whereas BAd7-209 and D97 were high-GPC lines (Table 1). Wheat plants were grown in the field with three biological replicates (10 rows each replicate) at the experimental field of the Sichuan Agricultural University over two wheat growing seasons (2016 and 2017) at Wenjiang (2016WJ and 2017WJ) and Chongzhou (2016CZ and 2017CZ). Individuals were planted 10-cm apart in a 2-m row with 30 cm between rows. A compound fertilizer [N: P: K (15: 15: 15)] was applied before sowing at a rate of 450 kg per hectare. Developing grains were sampled at 10, 14, 18, 22, 26, 30, 34, and 38 days after pollination (DAP) and snap-frozen in liquid nitrogen and then kept at −80°C for RNA-seq.

**TABLE 1:** Agronomic characteristics of BAd7-209, BAd23-1, CN16, and D97

Materials	Plant height (cm)	Number of spikelets per spike (no.)	Number of spikes (no.)	1,000-Kernel weight (g)
BAd7-209	128.25 ± 4.44b	17.60 ± 1.06b	8.80 ± 1.97a	47.8 ± 0.8a
BAd23-1	106.32 ± 5.37c	17.13 ± 1.41b	8.27 ± 1.98a	41.81 ± 1.6b
D97	143 ± 4.3a	11.50 ± 1.66c	7.82 ± 0.41b	19.15 ± 0.68c
CN16	83.0 ± 3.2d	18.60 ± 1.55a	8.5 ± 0.7a	43.4 ± 1.3ba

Note: The letters a, b, c, and d indicate the significant difference at 0.05 level with Turkey’s two-way test.

### Characterization of Agronomic Traits and Karyotype

Agronomic traits of BAd7-209 and BAd23-1 and their parents were measured as listed in Table 1. The traits of plant height (PH), number of spikelets per spike (NSp), and spike number per plant (SN) were averaged by 10 plants. The weight of 300 randomly selected seeds (GB/T 5519-2008, 2008) was recorded to represent the 1,000-kernel weight (TKW) as described by Wang et al. (2018).

At least 30 root-tip cells of BAd7-209 and BAd23-1 and their parents D97 and CN16 were observed for detection the chromosome number using the methods as described by Zhang et al. (2007).

### Single-Nucleotide Polymorphism (SNP) Genotyping

Genomic DNA from leaves was isolated using a plant genomic DNA kit (Tiangen Biotech, Beijing Co. Ltd. Beijing, China). Chip-based genotyping was conducted using the wheat 55 K SNP array (www.capit albio.com). The flanking sequence of each SNP was used to map onto the bread wheat reference sequence (https://urgi.versailles.inra.fr/download/iwgsc/IWGSC_RefSeq_Assemblies/v1.0/), using BLASTN with E-value threshold of 10^−10^ and a maximum mismatch of one base. SNP markers showed homozygous genotype among the parents, and advanced wheat lines were used to estimate D97 introgressions. The ratios of same SNP to the total SNPs scored between D97 and two advanced lines were computed using a sliding window of 10 Mb and step length of 1 Mb as described by Hao et al. (2019). Graphical representations were constructed using the R package ggplot2 (Wickham 2016).

### Quality Parameters Determination

Mature seeds were harvested for measurement of wheat quality parameters (Supplementary Table S1). Seeds of D97, CN16, BAd7-209, and BAd23-1 were conditioned to 14% moisture and milled using the Chopin CD1 AUTO (Renault, Boulogne-Billancourt, France) (Wang et al., 2018). Wet gluten content (WGC) was determined following the American Association of Cereal Chemists (AACC) method 38-12A. The sedimentation volume (SV) was measured following the AACC 44-15A (FOSS, Denmark). GPC (0.5 g) was recorded according to the AACC 39-10. Hydroscopic rate (HR) was measured following the AACC-54-21. All of the experiments were repeated three times.

### Grain Protein Component Measurement

Albumins, globulins, glutenins, and gliadins were extracted from mature grains using the methods as described by Duan and Zhao, 2004 and Wan et al. (2000) with slight modifications. Protein content was tested using Kjeldahl method (Kjeltec^TM^ 8400). Sodium dodecyl sulfate–polyacrylamide gel electrophoresis (SDS-PAGE) was used to separate the glutenins following the method of Hu et al. (2010) using the protein extraction buffer consisting of 62.5 mM Tris-HCl, pH 6.8, 10% (v/v) glycerol, 2% (w/v) SDS, 0.002% (w/v) bromophenol blue, and 1.5% (w/v) dithiothreitol (DTT).

### RNA-Seq Analysis

Total RNA was extracted using a plant RNA extraction kit v1.5 (Biofit Biotechnologies, Chengdu). The RNA concentration and integrity were assessed using an Qubit®RNA assay kit on a Qubit^®^ 2.0 Flurometer (Life Technologies, CA, United States) and an RNA Nano 6000 assay kit, respectively. RNAs from different grain developing stages were pooled in equi-molar concentrations (1 µg of RNA per sample) for cDNA library construction (three biological replicates per library). The sequencing libraries were completed using the NEBNext^®^ Ultra™ RNA Library Prep Kit for Illumina^®^ (NEB, United States) and then sequenced using the Illumina Hiseq platform (Novogene Bioinformatics Technology Co. Ltd. Beijing, China).

The adaptors, reads with more than 10% N, and reads with phred quality scored Q < 20 from RNA-seq raw data were removed using Trimmomatic (Bolger et al., 2014). The generated clean reads from each library were assessed using the Q20, Q30, and GC contents and aligned to Chinese Spring reference genome (https://urgi.versailles.inra.fr/blast_iwgsc/) using Hisat2 (Kim et al., 2019). FPKM (Elowitz et al., 2002; Jiang and Wong, 2009) was estimated to represent the expression level. DESeq was used for differential expression analysis as described by Wang et al. (2010). Genes with adjusted FDR <0.001 and |log_2_^FC^| ≥ 2 found by EBSeq (Leng et al., 2013) were considered as DEGs. Short Time-series Expression Miner (STEM) software (v1.3.11) was used to explore expression patterns of DEGs as described by Ernst and Bar-Joseph (2006) with log_2_ standardization, *p*-value ≤ 0.05, and |log_2_^FC^|≥2. Functional annotation of the DEGs was performed using Kyoto Encyclopedia of Genes and Genomes (KEGG) and Gene Ontology (GO) databases (Kanehisa et al., 2004).

### Prediction of Transcription Factors and Cis-elements

The putative plant TFs and cis-elements were predicted using iTAK software (Zheng et al., 2016) and PlantCARE (http://bioinformatics.psb.ugent.be/webtools/plantcare/html/) (Rombauts et al., 1999).

### Quantitative Real-Time PCR

Quantitative real-time PCR (qRT-PCR) was performed to validate the RNA-seq data. Gene-specific primers were designed using Primer3Plus software (http://www.primer3plus.com/) (Hung and Weng, 2016). Reactions were performed using the Bio-Rad CFX96 Real-Time PCR System (Bio-Rad, United States) in 10 µl of reaction volume containing 2 ng of cDNA, 5 µl of 1 × SYBR Premix ExTaq (TaKaRa), 0.5 µl (300 nM) of each primer, and ddH_2_O up to 10 µl (Wang et al., 2020). The 2^−ΔΔCT^ method (Schmittgen 2002) was used to quantify the gene expression with endogenous *GAPDH* as the internal reference.

### Statistical Analysis

Statistical analyses were conducted using SPSS version 22 software (SPSS Inc. Chicago, IL, United States). The morphological traits and quality parameters were compared by analysis of variance (ANOVA) complemented by Tukey’s test.

## Results

### Agronomic and Karyotype Characteristics

**FIGURE 1:**
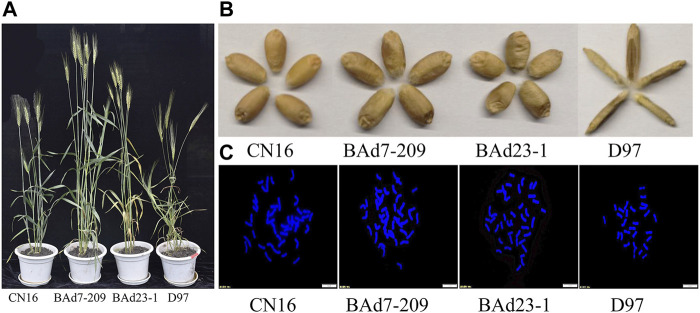
Agronomic traits and chromosome patterns of CN16, BAd7-209, BAd23-1, and D97. **(A)** Plants and **(B)** seeds of CN16, BAd7-209, BAd23-1, and D97. **(C)** The number of root-tip chromosomes; scale bar: 10 um.

The NSp of BAd7-209 and BAd23-1 were less than that of CN16 (*p* < 0.05), while higher than D97 (*p* < 0.05). The BAd7-209 resembled the BAd23-1 with respect to spike and SN. We have observed significantly differences in PH and TKW among BAd7-209, BAd23-1, and CN16 (Figure 1; Table 1). The D97 had significantly less NSp and TKW, while significantly higher PH than that of advanced wheat lines (Table 2).

**TABLE 2:** The major quality parameters in BAd7-209 and BAd23-1 and their parents.

Parameter	BAd7-209	BAd23-1	CN16	D97
Grain protein content (%)	15.8 ± 1.0b	13.8 ± 0.3c	12.6 ± 0.4d	23.70 ± 0.46a
Albumin (%)	3.31 ± 0.03b	3.045 ± 0.57b	3.455 ± 0.22b	6.32 ± 0a
Globumin (%)	0.27 ± 0.16b	0.245 ± 0.05b	0.245 ± 0.09b	2.19 ± 0a
gliadin (%)	3.06 ± 0.41b	2.3 ± 0.23c	2.8 ± 0b	6.77 ± 0a
glutenin (%)	6.34 ± 0.18b	4.78 ± 0.16c	5.025 ± 0.40b	6.48 ± a
Wet glutein content (%)	39.49 ± 0.55a	24.89 ± 0.41b	28.01 ± 0.51b	ND
Sedimentation volume (ml)	41.3 ± 0.42a	22.5 ± 0.71c	26.6 ± 0.57b	ND
Hydroscopic rate (%)	64.6 ± 2.55a	55.8 ± 1.41a	53.6 ± 3.39a	ND
Stabilization time (min)	7.93 ± 0.11a	3.9 ± 0.14b	1.61 ± 0.08c	ND

Note: ND, not determined; the letters a, b, c, and d indicate the significant difference at 0.05 level with Turkey’s two-way test.

The BAd7-209 had higher SV (41.3%), WGC (39.49%), HR (64.6%), and stabilization time (7.93%) than those of BAd23-1 and CN16. The GPC in D97 was highest (23.70%), followed by BAd7-209 (15.8%), BAd23-1 (13.8%), and CN16 (12.6%) (Table 2). The WGC, SV, HR, and GPC levels of BAd7-209 were higher than the strong-gluten wheat and those of BAd23-1 were between weak-gluten and medium-gluten wheat.

Cytological observations indicated that both BAd23-1 and BAd7-209 have 42 chromosomes (Figure 1C), which reached the genetic background of common wheat. SNP genotyping analyses revealed 150 and 108 potential D97 segments on chromosomes of BAd23-1 and BAd7-209. The number of CN16 segments on BAd7-209 and BAd23-1 chromosomes was 212 and 232 (Supplementary Figure S1).

### Grain Protein Component and Glutenin Subunits

The total amount of glutenins, gliadins, albumins, and globulins in D97 was higher than those of CN16. There were no differences in albumin and globulin contents between BAd23-1 and BAd7-209. The glutenin and gliadin contents of BAd7-209 were significantly higher than those of BAd23-1 (Table 2).

**FIGURE 2:**
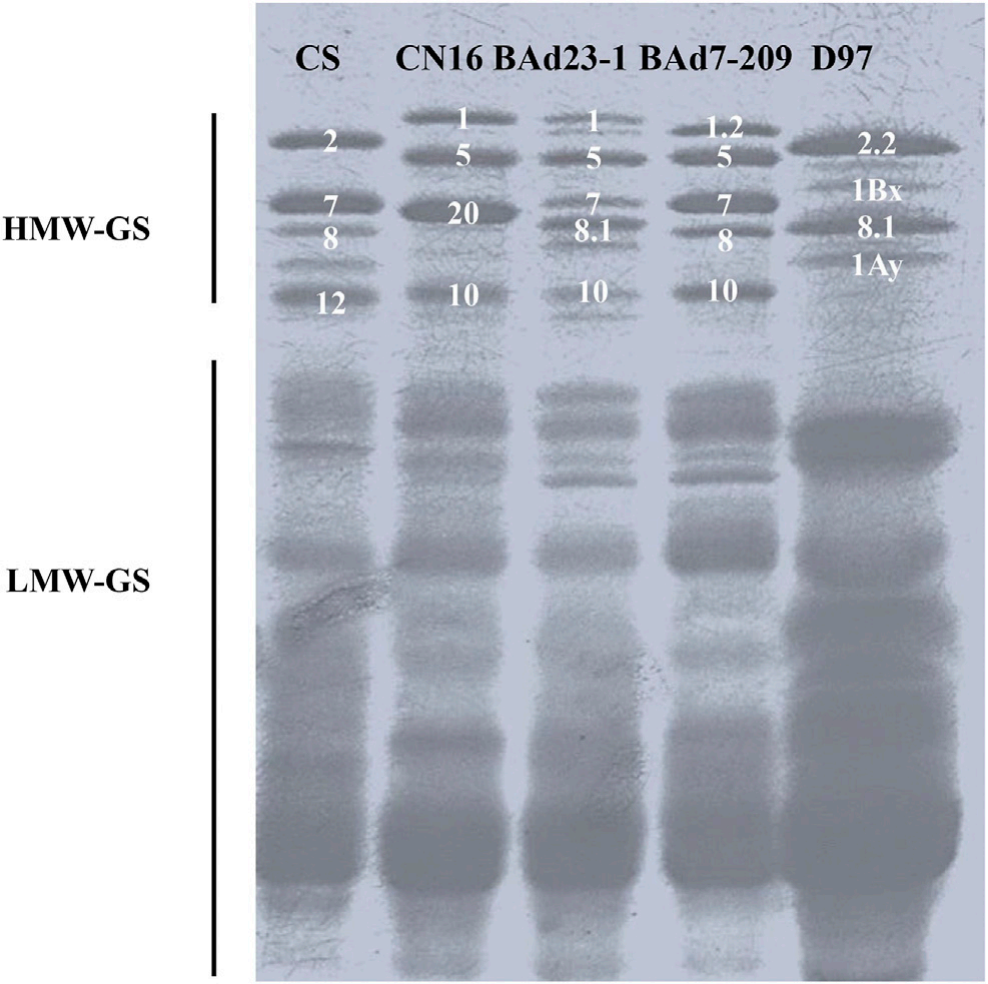
Separation of glutenin proteins from BAd7-209 and BAd23-1 and their parents by SDS-PAGE and Chinese Spring (CS) as the references.

SDS-PAGE analysis showed different high-molecular-glutenin subunits (HMW-GSs) in CN16 (1Ax1, 1Dx5, 1Dy10, and 1Bx+1By20) and D97 (1Ax2.2, 1Bx, 1By8.1, and 1Ay). The BAd7-209 possessed five HWM-GSs (1Ax1.2, 1Bx7 + 1By8, and 1Dx5 + 1Dy10), which were consistent with previous reports (Jiang et al., 2017; Xiang et al., 2019). The BAd23-1 had five HMW-GSs, including 1Bx7 and 1Dx5+1Dy10 that are identical to BAd7-209, and one different 1Ax subunit (1Ax1) and one 1By subunit (1By8.1) (Figure 2).

The expression levels of 1Ax and 1By in high-GPC group (D97 + BAd7-209) were higher than those of low-GPC group (CN16 + BAd23-1). The composition of low-molecular-glutenin subunits (LMW-GSs) in CN16, BAd7-209, and BAd23-1 was similar. The wild emmer D97 showed less LWM-GS subunits probably due to the absence of the D genome. The expression levels of LMW-GSs in high-GPC group (D97 + BAd7-209) were higher than those of low-GPC group (CN16 + BAd23-1) (Figure 2).

### Analysis of RNA-Seq Data

RNA-seq generated 189.93 million raw reads from developing grains of BAd7-209, BAd23-1, CN16, and D97 pools. A total of 94.96 million clean reads were retained and further mapped to the *T. aestivum* cDNA database (IWGSC1.0). The mapping ratios ranged from 79.42% to 84.33%, 86.92% to 88.12%, 85.76% to 89.33%, and 79.15% to 80.89% in BAd7-209, BAd23-1, CN16, and D97, respectively. The GC contents among replicates were almost identical, and the Q30 was over 90% in each library (Supplementary Table S2). These results demonstrate that the RNA-seq data were qualified for subsequent analysis.

### Differential Expression Analysis

**FIGURE 3:**
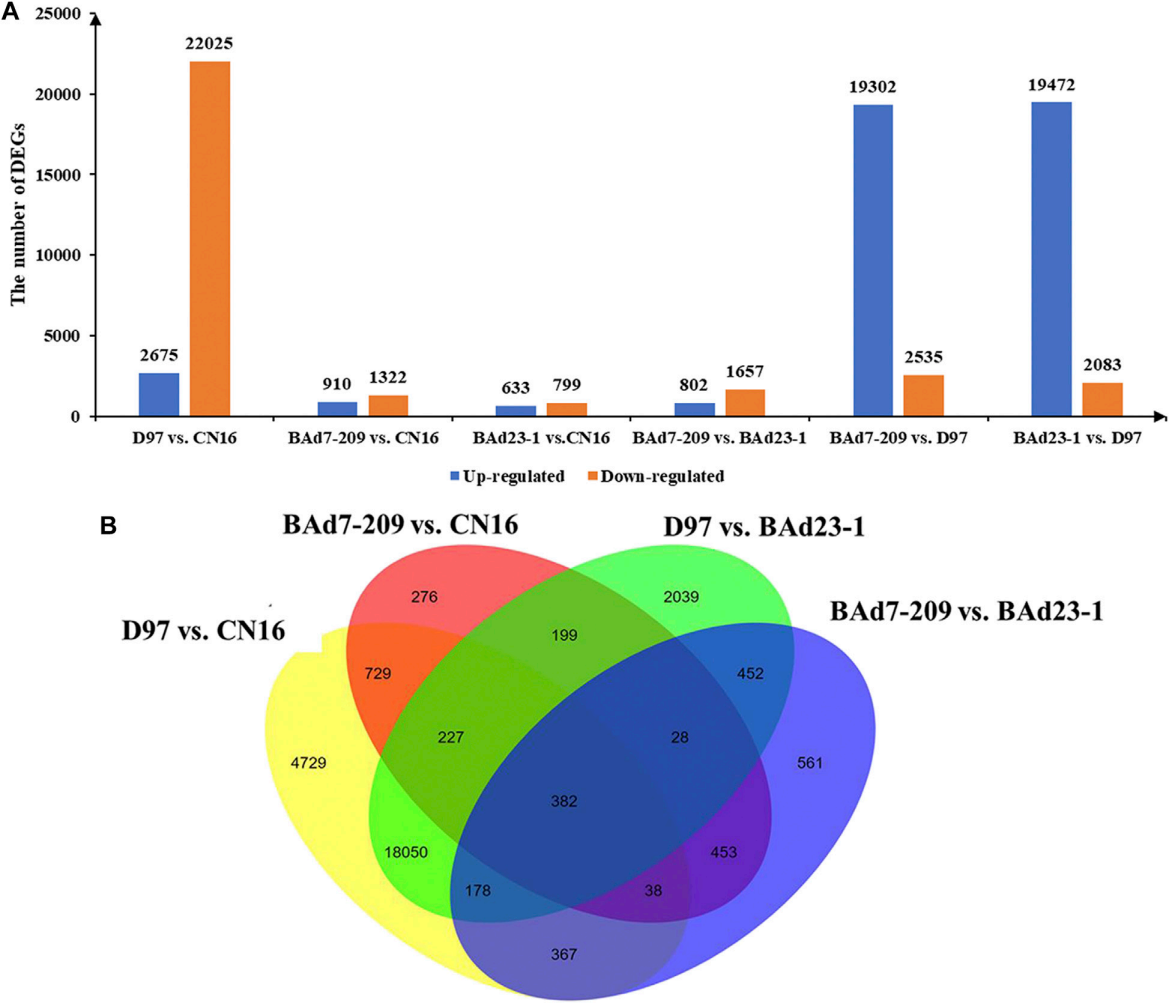
The histograms and Venn diagrams of DEGs in different comparisons. **(A)** Up- and downregulated DEGs in BAd7-209 and BAd23-1 and their parents. **(B)** The Venn diagram of DEGs in the high-GPC (D97 + BAd7-209) vs. low-GPC (CN16 + BAd23-1) groups.

**FIGURE 4:**
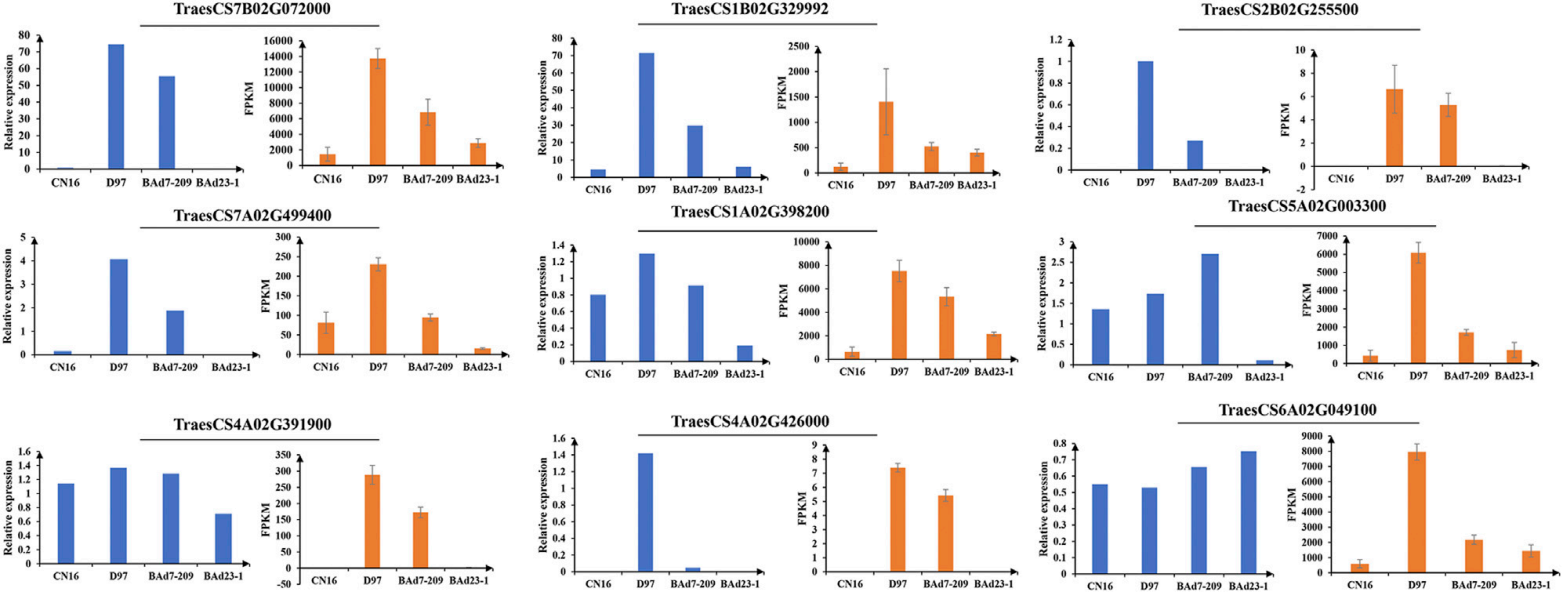
Validation of DEGs by qRT-PCR.

In total, 62,404 genes mapped to IWGSC1.0 genome were expressed (FPKM≥1) in D97, CN16, BAd7-209, and BAd23-1 libraries. A total of 24,700, 2,332, 21,555, and 2,459 DEGs (|log_2_^FC^|≥2) were found in D97 vs. CN16, BAd7-209 vs. CN16, D97 vs. BAd23-1, and BAd7-209 vs. BAd23-1, respectively (Figure 3A). The comparison between the high-GPC group (D97 + BAd7-209) and low-GPC group (CN16 + BAd23-1) revealed 382 DEGs (Figure 3B), of which 148 genes were common expressed, 34 genes only expressed in D97 and BAd7-209, whereas 56 genes only expressed in CN16 and BAd23-1. Nine DEGs encoding HWM-GS, alpha-gliadin, nodulin protein, trypsin inhibitor, purothionin, and pre-mRNA-splicing factor proteins were selected to perform qRT-PCR (Supplementary Table S3). The expression changes of seven DEGs were quite consistent with those obtained from the RNA-seq (Figure 4, Supplementary Table S3).

**FIGURE 5:**
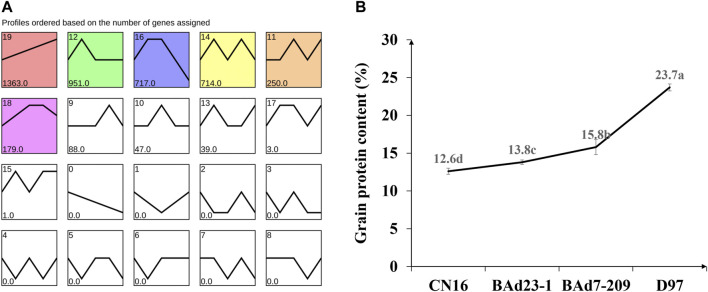
Temporal expression patterns of DEGs **(A)** and grain protein content in CN16, BAd23-1, BAd7-209, and D97 **(B)**. Small letters a, b, c, and d indicate the significant difference at 0.05 level with Tukey’s two-way test.

All genes were further analyzed with the STEM software (v1.3.11) (Ernst and Bar-Joseph, 2006) to obtain the temporal expression patterns. Nineteen expression profiles were clustered, and seven profiles (9, 10, 0, 19, 4, 7, and 6) were regarded as significantly changed (*p* ≤ 0.05) (Figure 5A). The profile 19 had similar tendency to the GPC in advanced lines and the parents (Figure 5B), indicating the positive role in regulation of grain protein accumulation.

### The Functional Annotation of DEGs

**FIGURE 6:**
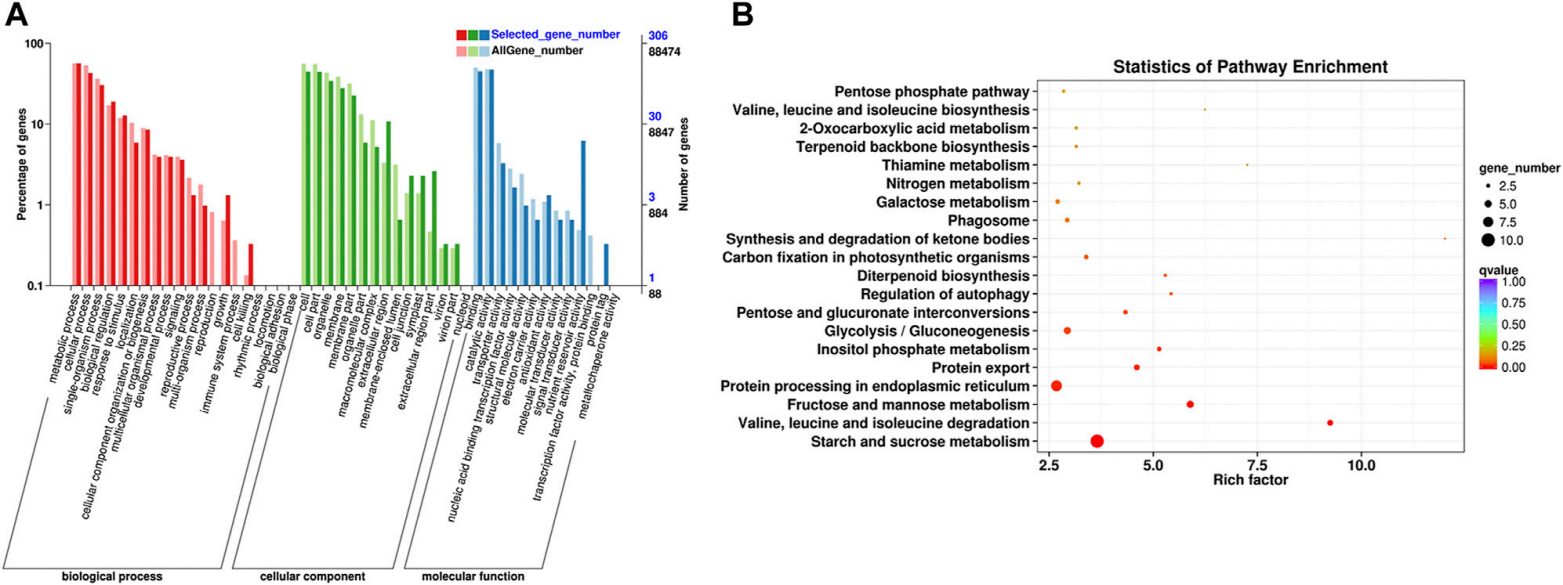
Functional annotation of the DEGs based on GO and KEGG pathway enrichment analyses. **(A)** GO classification of the DEGs. **(B)** KEGG classification of the DEGs.

A total of 459 DEGs in profile 19 were subjected to GO and KEGG analyses. On the basis of GO terms for those genes, three categories can be classified: biological process, cellular component, and molecular function. The GO term metabolic process (GO: 0008152, 172 DEGs), cellular process (GO: 0009987, 131 DEGs), and single-organism process (GO: 0044699, 93 DEGs) were highly enriched in biological process; those of cell (GO: 0005623, 137 DEGs), cell part (GO: 0044464, 136 DEGs), and organelle (GO: 0043226, 105 DEGs) were dominant in cellular component; and those of binding (GO: 0005488, 138 DEGs), catalytic activity (GO: 0003824, 145 DEGs), and nutrient reservoir activity (GO: 0045735, 19 DEGs) were primary in molecular function (Figure 6A, Supplementary Table S4).

KEGG pathway enrichment analysis showed that starch and sucrose metabolism (ko00500, 10 DEGs), protein processing in ER (ko04141, eight DEGs), fructose and mannose metabolism (ko00051, five DEGs), glycolysis/gluconeogenesis (ko00010, five DEGs), protein export (ko03060, four DEGs), inositol phosphate metabolism (ko00562, three DEGs), pentose and glucuronate interconversions (ko00040, three DEGs), valine, leucine, and isoleucine degradation (ko002800, one DEG) were significantly enriched pathways (Figure 6B, Supplementary Table S5). Eight DEGs in pathway of protein processing in ER are related to protein transport protein Sec61 (Sec61, 3 genes), heat shock protein (HSP, 3 genes), SKP1-like protein (SKP1, 1 gene), and E3 ubiquitin-protein ligase RMA3 (RMA3, 1 gene). Four genes in pathway of protein export are related to Sec61 (3 genes) and signal recognition particle 54 kDa protein 2-like (SRP, 1 gene).

### Characterization of DEGs Encoded Gluten and TFs

Twelve out of 459 genes in profile 19 were related to α/β-gliadins (six genes), γ-gliadins (three genes), LMW-GSs (two genes), and HWM-GSs (one gene) (Table 3). All these genes were upregulated in the high-GPC group compared to the low-GPC group. Twenty-eight genes belonging to 16 TF families, such as NAC, MYB, GARP-G2-like, bZIP, and C2H2, were identified (Table 4). To understand the expression patterns, the expression levels of TFs in different tissues sampled at different time points were retrieved from public available RNA-seq data of IWGSC (Thomas et al., 2014). We found that seven genes (e.g., *TabZIP8700*, *TaC2H20600*, and *TaMYB0300*) were expressed in different tissues at different time points. Ten genes (e.g., *SPA*, *TaC3H8000*, *TaGAMyb*, and *TaNAC019*) were only expressed in grains. Two genes (*TaMYB7800* and *TaNAC9500*) were root specific. The others were not detected in all tissues (Supplementary Table S6).

**TABLE 3:** Cis-element in the promoter region of grain protein gene.

Geneid	FPKM				Cis-element				
	CN16	D97	BAd7-209	BAd23-1	GCN4	P-box	AACA	ACGCAA/G	Description
TraesCS6A02G049100	590	7,951.33	2,176.12	1830.75	+	+	−	+	α/β-gliadin
TraesCS6A02G049400	173.93	2,891.6	627.41	880.7	+	+	−	−	α/β-gliadin
TraesCS6A02G049500	18.94	797.11	151.39	179.42	+	−	−	−	α/β-gliadin
TraesCS6B02G065600	63.88	905.52	420.37	370.42	+	−	−	−	α/β-gliadin
TraesCS6B02G065749	112.64	2,186.8	573.67	784.51	+	−	−	−	α/β-gliadin
TraesCS6B02G065800	229.42	1,581.61	806.87	914.95	+	−	−	−	α/β-gliadin
TraesCS1A02G007344	614.17	5,175.81	2,263	1,685.48	+	+	+	+	γ-gliadin
TraesCS1A02G007400	485.43	10,810.48	1783.58	1,352.3	+	+	−	+	γ-gliadin
TraesCS1A02G007405	98.74	2,160.94	443.16	346.52	+	+	−	+	γ-gliadin
TraesCS1B02G329992	211.76	1,405.30	523.24	452.47	−	+	+	+	HMW-Glutenin
TraesCS1A02G007934	34.05	452.88	234.47	110.35	+	+	−	−	LMW-Glutenin
TraesCS1A02G008000	717.6	4,225.24	3,249.7	2,760.87	+	+	−	−	LMW-Glutenin
TraesCS1A02G066100	95.21	1786.88	835.5	357.43	+	−	+	−	12S globulin
TraesCS1A02G317500	226.07	3,879.86	1,045.96	769.24	+	−	−	−	19 kDa globulin
TraesCS1B02G330000	201.04	2,660.14	1,076.59	816.59	+	−	−	−	19 kDa globulin
TraesCS1A02G007700	881.24	12,190.49	3,893.48	2,723.56	+	+	+	−	Avenin-like
TraesCS7A02G035200	11.92	151	45.15	36.84	+	+	−	−	Avenin-like

**TABLE 4:** The list of TFs identified from the 459 DEGs in profile 19.

GeneName	GeneID	TF family	FPKM			
			CN16	BAd23-1	BAd7-209	D97
TaAUX0996	*Triticum*_*aestivum*_newGene_30,996	AUX/IAA	0	0	0.03	1.13
TaB30200	TraesCS3B02G530200	B3	0.01	0.54	0.14	3.37
SPA	TraesCS1A02G329900	bZIP	5.32	9.01	12.48	23.15
TabZIP8700	TraesCS3A02G378700	bZIP	1.08	1.55	1.93	4.86
TaC2H20600	TraesCS5B02G490600	C2H2	0.31	1.79	2.43	5.27
TaC3H8000	TraesCS2B02G138000	C3H	0.8	1.99	4.5	9.65
TaCSD9500	TraesCS6A02G069500	CSD	23.33	57.2	84.36	115.58
TaDBP8500	TraesCS6B02G398500	DBP	8.3	17.93	27.02	37.26
TaFAR079	*Triticum*_*aestivum*_newGene_56,079	FAR1	0.26	0.52	0.84	1.62
TaGA8200	TraesCS6B02G138200	GARP-G2-like	0.03	0.04	0.13	0.97
TaGA1000	TraesCS7A02G411000	GARP-G2-like	0.04	0.19	0.94	1.16
TaGR5900	TraesCS4A02G185900	GRAS	0.44	0.88	1.28	2
Taju6200	TraesCS5A02G166200	Jumonji	0	0.84	1.44	1.59
TaMA1500	TraesCS4B02G351500	MADS-M-type	1.41	1.36	2.8	5.67
TaMYB0300	TraesCS2A02G370300	MYB	7.12	12.29	17.97	28.99
TaMYB2000	TraesCS2B02G252000	MYB	1.45	1.63	2.49	4.86
TaMYB7800	TraesCS2B02G387800	MYB	0.75	1.96	4.3	5.94
TaGAMyb	TraesCS3A02G336500	MYB	3.54	8.04	12.93	16.15
TaNAC7700	TraesCS3A02G377700	NAC	0.19	0.26	0.5	1.31
TaNAC2900	TraesCS3B02G092900	NAC	0.43	1.77	4.14	6.29
TaNAC2400	TraesCS7A02G152400	NAC	0.52	2.63	4.18	6.16
TaNAC4700	TraesCS7A02G194700	NAC	6.33	32.32	37.62	56.61
TaNAC9500	TraesCS7A02G349500	NAC	0.08	0.25	0.18	0.55
TaNAC9100	TraesCS7A02G569100	NAC	5.76	23.67	34.2	56.1
TaNAC9300	TraesCS7A02G569300	NAC	0.04	0.54	1.25	3.03
TaNAC6300	TraesCS7B02G056300	NAC	3.18	11.97	30.07	64.68
TaNF6700	TraesCS7A02G336700	NF-YC	52.73	113.72	199.94	192.12
Ta3500	TraesCSU02G193500	Others	0	0	0.03	1.82

## Discussion

GPC is an important quality trait in common wheat. The wild emmer gene *GPC-B1* positively impacts protein, Zn, and Fe in wheat grain (Uauy et al., 2006). In the current study, two sister wheat lines BAd7-209 and BAd23-1 derived from wild emmer showed contrasting GPC, while both of them did not contain the *Gpc-B1*. Processing quality parameters of BAd7-209 were significantly higher than those of BAd23-1. These two lines had genetic background of common wheat and introgression segments from wild emmer D97. Our results indicate the presence of other wild emmer gene(s) that contributed to high GPC in BAd7-209.

Previous reports revealed that the components and expression levels of glutenins and gliadins could affect GPC (Xi and Zheng, 2011; Jiang et al., 2017; Wang et al., 2018; Xiang et al., 2019; Shen et al., 2020). In this study, similar glutenins subunits were identified in BAd7-209 and BAd23-1, whereas BAd7-209 had significant higher amount of glutenins and gliadins than BAd23-1. Nineteen DEGs including gliadin (nine genes), glutenin (three genes), globulin (three genes), avenin-like (three genes), and serpin (two genes) were enriched in the GO term nutrient reservoir activity (GO: 0045735). These genes were upregulated in the high GPC (D97 + BAd7-209) vs. the low-GPC (CN16 + BAd23-1) groups. Previous reports revealed that overexpression gliadin and glutenin genes could increase GPC in wheat (Guo et al., 2015; Li et al., 2019) and rice (Cho et al., 2019). Serpin genes were positively associated with wheat grain development (Benbow et al., 2019) and seed germination (Dong et al., 2015). The results in this study together with previous reports (Jiang et al., 2017; Wang et al., 2018; Xiang et al., 2019; Gong et al., 2021) show that the GPC is closely associated with the expression levels of glutenin and gliadin genes in wheat.

**FIGURE 7:**
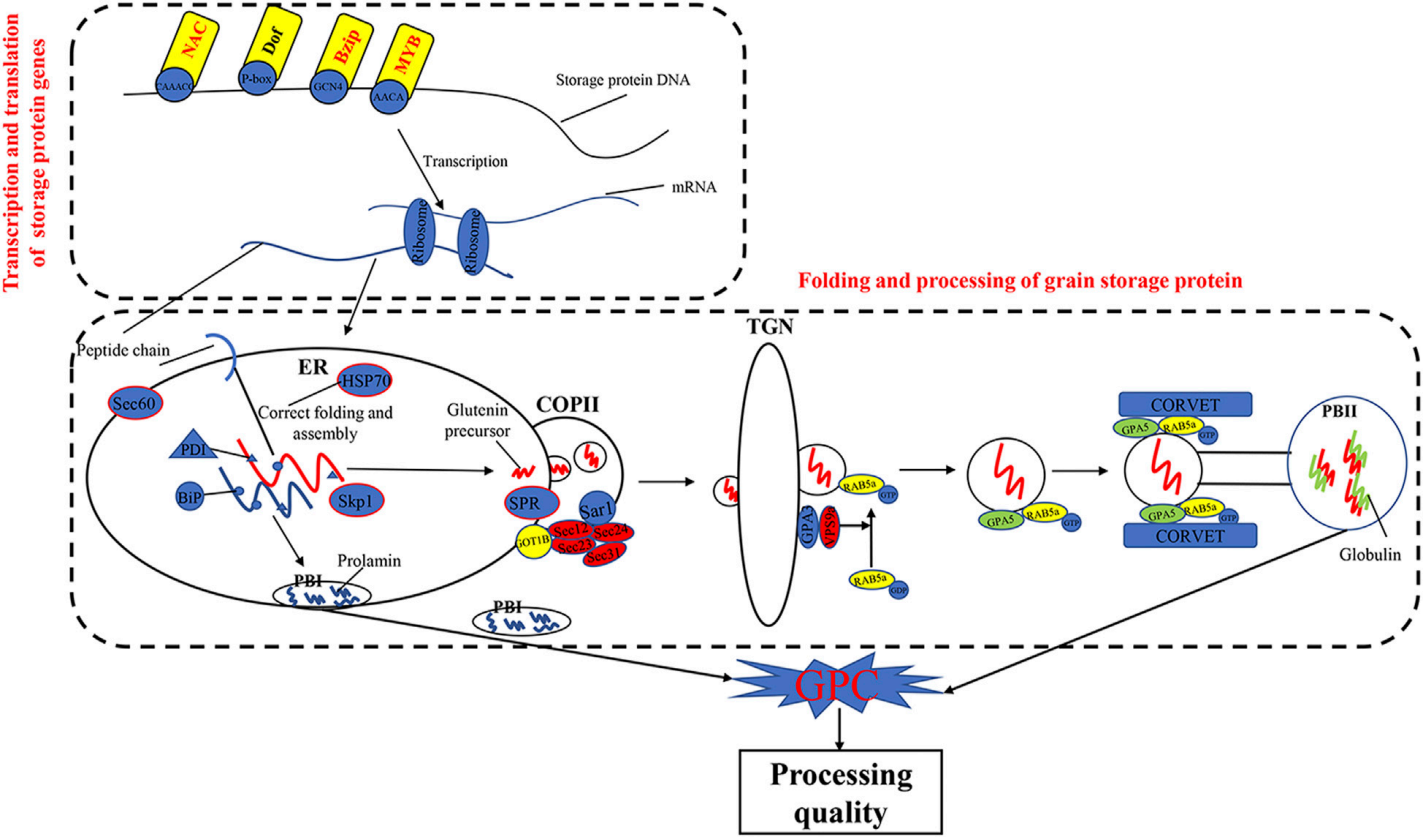
The synthesis and regulation pathways of grain storage protein.

The KEGG pathways of processing in ER (ko04141) and protein export (ko03060) were previously reported to play important roles in the folding and maturation processing of grain storage protein in cereals (Crofts et al., 1998; Foresti et al., 2003; Strasser, 2018; Yu et al., 2018; Yu et al., 2020; Wang et al., 2016; Zhu et al., 2014) (Figure 7). In the present study, we found that DEGs, such as HSP, Sec61, SKP1, RMA3, and SRP, were significantly enriched in the two pathways (ko04141 and ko03060). Previous studies have shown that Hsp70 (Dupont et al., 1998) was a key chaperone for the processing and polymerization processes of grain storage proteins in ER. The Sec proteins (e.g., *Sec13*, *Sec23*, *Sec24*, and *Sec31*) were involved in transferring proteins from the ER to the Golgi apparatus (D'Arcangelo et al., 2013). SKP1 protein is a key member of the SCF (SKP–cullin–F-box protein) E3 ligase complex and mediates the regulation of plant ABA sensitivity (Hu et al., 2013; Rao et al., 2018). In wheat plants, ABA treatment could accelerate nitrogen remobilization from vegetative organs to grains (Saeed, 2012; Xie et al., 2004). In addition, the SRP proteins are essential for protein translocation across the ER (Walter and Blobel, 1982). These results demonstrated that the pathways of protein processing in ER and protein export and related DEGs are important in wheat grain protein accumulation.

TFs were involved in transcriptional regulation of gluten genes through binding their cis-elements in promoters (Albani et al., 1997; Xi and Zheng, 2011). For example, the bZIP TFs could bind GCN4 motifs of gliadin and glutenin genes in maize (*O2*) (Schmidt et al., 1992; Vicente-Carbajosa et al., 1998). The Dof TFs (*PBF*) could activate α-gliadin and *Glu-1* genes in wheat (Zhu et al., 2018) by binding P-box elements. MYB TFs were specifically bound to the AACA motifs of gluten genes in rice (*OsMYB5*) (Suzuki et al., 1998), barley (*HvGAMYB*, *HvMYBS2*, and *HvMCB1*) (Gubler et al., 1995; Diaz et al., 2002), and wheat (*TaGAMyb*) (Guo et al., 2015). The *TaNAC019* activated the expression of HMW-GS gene by binding to the [AT]NNNNNNNNNNN [ATC][CG]A [CA]GN [ACT]A motif in the promoter region (Gao et al., 2021). In the present study, we have identified 28 TFs, such as NAC, MYB, GARP-G2-like, bZIP, and C_2_H_2_, from the high-GPC and low-GPC groups. Some TFs (e.g., *TaC3H8000*, *TaNAC2400*, *TaNAC4700*, and *TaNF6700*) were specifically expressed in grains. In addition, we have identified either NAC (ACGCAA/G), MYB (AACA), bZIP (GCN4), or Dof (P-box) motifs in the promoters of gluten genes that were differentially expressed in the high-GPC vs. low-GPC groups. Taken together, our results indicate that some TFs are involved in regulation of gluten and gliadin genes that contributed to the GPC accumulation in BAd7-209 (Figure 7).

## Conclusion

In the present study, we have characterized the agronomic, cytological, grain protein characteristics, and transcriptomic profile of two advanced wheat lines (BAd7-209 and BAd23) from a cross between CN16 and D97. We found that BAd7-209 and BAd23 had genetic background of common wheat and introgression segments from wild emmer D97. The two advanced lines had contrasting GPC, while both absence of the functional *GPC-B1*. BAd7-209 had superior processing quality parameters and higher amount of glutenins and gliadins than those of BAd23-1, while their glutenin and gliadin subunits were similar. RNA-seq revealed that the contrasting GPC in BAd7-209 and BAd23-1 may be closely associated with the expression levels of glutenin and gliadin genes which regulated by TFs. The protein processing in ER and protein export pathways and related DEGs are important in wheat grain protein accumulation.

## Data Availability

The original contributions presented in the study are publicly available in NCBI under accession number PRJNA777016.
