# Supplementary material for: Comparison of the Agronomic, Cytological, Grain Protein Characteristics, as Well as Transcriptomic Profile of Two Wheat Lines Derived From Wild Emmer
Source: Front Genet. 2022 Jan 28;12:804481. doi: 10.3389/fgene.2021.804481 (PMC8831750; doi:10.3389/fgene.2021.804481)
Supplement: Supplementary file 1 [file DataSheet1.zip › Data Sheet 1/Supplementary_Material.docx]

Supplementary Material

**Supplementary Tables (Table S1-S3)**

| **Table S1** The quality indicators of wheat（GB/T17320-2017). | | | | | | | | |
| --- | --- | --- | --- | --- | --- | --- | --- | --- |
| Project | | Strong-gluten | | Medium-strong gluten | | Medium-gluten | | Weak-gluten |
| Protein content (%) | | ≥ 14.0 | | ≥ 13.0 | | ≥ 12 | | < 12.0 |
| Wet gluten content (%) | | ≥ 30.5 | | ≥ 28.5 | | ≥ 24 | | < 24 |
| Sedimentation volume (mL) | | ≥ 40 | | ≥ 35 | | ≥ 30 | | < 30 |
| Hydroscopic rate (%) | | ≥ 60 | | ≥ 58 | | ≥ 55 | | < 55 |
| Stabilization time (min) | | ≥ 10.0 | | ≥ 7.0 | | ≥ 3.0 | | < 3.0 |
| **Table S2** Summary of RNA-seq data in BAd7-209, BAd23-1, and their parents.   \| Sample name \| Biological repetition \| Raw reads \| Clean reads \| Mapping to CS ratio (%) \| Q30 (%) \| GC content (%) \| \| --- \| --- \| --- \| --- \| --- \| --- \| --- \| \| CN16 \| R1 \| 52,228,646 \| 26,114,323 \| 89.06 \| 91.57 \| 55.17 \| \|  \| R2 \| 46,132,576 \| 23,066,288 \| 85.76 \| 91.08 \| 55.36 \| \|  \| R3 \| 48,099,354 \| 24,049,677 \| 89.33 \| 90.88 \| 56.57 \| \|  \| Average \| 48,820,192 \| 24,410,096 \| 88.05 \| 91.18 \| 55.7 \| \| D97 \| R1 \| 42,955,406 \| 21,477,703 \| 79.15 \| 91.01 \| 55.31 \| \|  \| R2 \| 44,698,114 \| 22,349,057 \| 79.44 \| 89.55 \| 54.77 \| \|  \| R3 \| 47,193,938 \| 23,596,969 \| 80.89 \| 89.9 \| 53.73 \| \|  \| Average \| 44,949,152.67 \| 22,474,576.33 \| 79.83 \| 90.15 \| 54.6 \| \| BAd7-209 \| R1 \| 44,232,490 \| 22,116,245 \| 81.72 \| 90.94 \| 54.41 \| \| R2 \| 49,989,052 \| 24,994,526 \| 79.42 \| 90.56 \| 53.92 \| \| R3 \| 43,183,276 \| 21,591,638 \| 84.33 \| 91.33 \| 55.75 \| \| Average \| 45,801,606 \| 22,900,803 \| 81.82 \| 90.94 \| 54.69 \| \| BAd23-1 \| R1 \| 48,907,908 \| 24,453,954 \| 87.49 \| 90.77 \| 56.06 \| \| R2 \| 54,882,836 \| 27,441,418 \| 88.12 \| 91.41 \| 55.35 \| \| R3 \| 47,276,720 \| 23,638,360 \| 86.92 \| 90.86 \| 53.32 \| \| Average \| 50,355,821.33 \| 25,177,910.67 \| 87.51 \| 91.01 \| 54.91 \| \| Total \|  \| 189,926,772 \| 94,963,386 \|  \|  \|  \|   **Table S3** List of primers used for qRT-PCR analysis. | | | | | | |  |  |
| GeneID | Forward (5'-3') | | Reverse (5'-3') | | Annotation | |  |  |
| TraesCS7A02G499400 | CATCCCCATCAACAACATCATCG | | CAGGCCTTTAACAACAAGTCCAG | | Protein transport protein Sec61 | |  |  |
| TraesCS1B02G329992 | CCCATACCATGTTAACACAGAGC | | CTATCACTGGCTAGCCGACAAT | | HMW glutenin subunit 1By22* | |  |  |
| TraesCS7B02G072000 | GTCCAAGTCTAGCATCACCC | | AAGTTTGCTGTGCGACATAC | | Alpha-amylase/trypsin inhibitor CM2 | |  |  |
| TraesCS1A02G398200 | AGTCCAACTCAGATGAACCAGAC | | TCACCACACTGTTCCACATAGAG | | beta purothionin | |  |  |
| TraesCS5A02G003300 | CTAGCTTTCCTTGCTCTGGTAGT | | CCGATCCATAACATAATCGCTGC | | GSP-1 Grain Softness Protein | |  |  |
| TraesCS4A02G391900 | GAATGGCCTACTTCATCGTCG | | TAGTTGAAGGAGGTGTTCTTGAGG | | Early nodulin-93 | |  |  |
| TraesCS4A02G426000 | CAAAAACTGACAAGGGGAAGGTC | | CTGAACCCTTGCTCCTTGATTTC | | pre-mRNA-splicing factor cwf23-like | |  |  |
| TraesCS2B02G255500 | CCCGGATTGGATTGGACTAGATT | | GGATATCTTCTGGAAGCTGCAGA | | early nodulin-like protein | |  |  |
| TraesCS6A02G049100 | GCAACAATATCCATTAGGCCAGG | | CGCTAGGTTCCTTATTTCCTCGA | | alpha-gliadin | |  |  |

**Supplementary Figures**


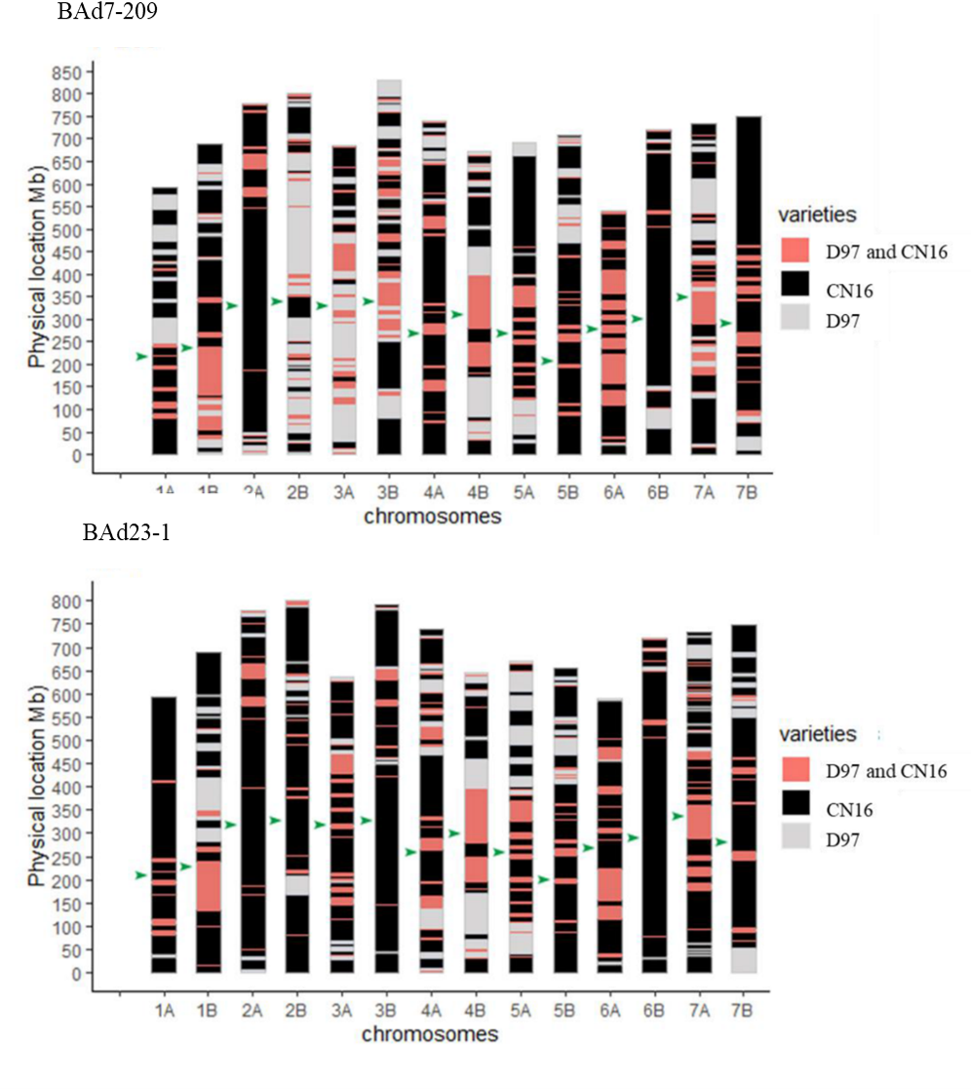


Fig. S1 the graphical genotypes of BAd7-209 and BAd23-1 with the fragments from the wild emmer D97 and common wheat CN16, respectively. The green arrowheads indicate the centromeres. The colored boxes refer to segments from D97 and CN16.
